# Loss of the fragile X mental retardation protein causes aberrant differentiation in human neural progenitor cells

**DOI:** 10.1038/s41598-018-30025-4

**Published:** 2018-08-02

**Authors:** Naohiro Sunamura, Shinzo Iwashita, Kei Enomoto, Taisuke Kadoshima, Fujio Isono

**Affiliations:** Asubio Pharma Co., Ltd., 6-4-3 Minatojima-minamimachi, Chuo-ku, Kobe 650-0047 Japan

## Abstract

Fragile X syndrome (FXS) is caused by transcriptional silencing of the *FMR1* gene during embryonic development with the consequent loss of the encoded fragile X mental retardation protein (FMRP). The pathological mechanisms of FXS have been extensively studied using the *Fmr1*-knockout mouse, and the findings suggest important roles for FMRP in synaptic plasticity and proper functioning of neural networks. However, the function of FMRP during early development in the human nervous system remains to be confirmed. Here we describe human neural progenitor cells (NPCs) as a model for studying FMRP functions and FXS pathology. Transcriptome analysis of the NPCs derived from *FMR1*-knockout human induced pluripotent stem cells (iPSCs) showed altered expression of neural differentiation markers, particularly a marked induction of the astrocyte marker glial fibrillary acidic protein (GFAP). When induced to differentiate, FMRP-deficient neurons continued to express GFAP, and showed less spontaneous calcium bursts than the parental iPSC-derived neurons. Interestingly, the aberrant expression of GFAP and the impaired firing was corrected by treatment with the protein kinase inhibitor LX7101. These findings underscore the modulatory roles of FMRP in human neurogenesis, and further demonstrate that the defective phenotype of FXS could be reversed at least partly by small molecule kinase inhibitors.

## Introduction

Fragile X syndrome (FXS) is the most common inherited form of intellectual disability frequently associated with autism^1,2^. FXS is an X-linked monogenic disorder caused by a loss of function of the fragile X mental retardation protein (FMRP) encoded by the *FMR1* gene^3,4^. In most patients, expansion of the CGG trinucleotide repeats within the 5′-UTR of the *FMR1* gene results in widespread methylation and transcriptional silencing of the gene early in embryogenesis^5^. FMRP is an RNA binding protein abundantly expressed in the brain, and is enriched in actively translating polyribosomal fractions^6^. FMRP interacts with a specific set of target mRNAs including pre- and post-synaptic proteins important for plasticity, and presumably controls their local translation^7–9^. For example, FMRP binds the coding region of its target mRNAs and reversibly stalls ribosomes on the targets, thereby repressing their translation^10^. Dysregulated protein translation caused by the loss of functional FMRP could perturb neuronal development and function by disrupting synaptic maturation and plasticity.

*Fmr1* knockout *(Fmr1*-KO) mice have been extensively studied as a model for FXS, because they recapitulate many aspects of the FXS phenotype such as locomotive hyperactivity, altered social interactions, hypersensitivity to sensory stimuli, and mild learning deficits^11,12^. In addition, neurons derived from *Fmr1-KO* mice show an increased number of elongated and thin spines, the hallmark of dendritic abnormality in FXS^13–15^. Mechanistic studies revealed many molecular signalling cascades involved in neuronal development or synaptic plasticity function abnormally in the *Fmr1*-KO mouse, and provided potential targets for therapeutic intervention^16^. The metabotropic glutamate receptor (mGluR) theory is the most widely accepted hypothesis that enhanced signalling downstream of post-synaptic group 1 mGluR (mGluR1 and mGluR5) contributes to the molecular, electrophysiological, and behavioural dysfunction in *Fmr1*-KO mouse^17^. Indeed, using pharmacological and genetic approaches, the FXS-associated phenotypes have been ameliorated by inhibiting mGluR in mouse models^18,19^. However, despite promising preclinical data, clinical trials with mGluR antagonists have not been successful, highlighting the necessity of further studies using human materials^20^.

Although nervous system research using human materials has traditionally been difficult, human pluripotent stem cells now provide useful models to study molecular mechanisms of neuronal diseases, and allow us to re-evaluate the observations in animal studies. In order to model FXS, patient-derived neural progenitor cells (NPCs), embryonic stem cells (ESCs), and induced pluripotent stem cells (iPSCs) have been established and analysed^21–27^. Initial research using NPCs isolated from foetal tissue of FXS patients showed abnormalities in culture^21,22^. More detailed analyses in neurons differentiated from patient-derived ESCs or iPSCs showed aberrant gene expression profiles during neurogenesis as well as morphological and functional abnormalities. Notably, neurons differentiated from patient-derived ESCs and iPSCs showed an immature phenotype with impaired excitability and synaptic connectivity^23–27^. For example, Telias *et al*. found the fewer synaptic vesicles and lack of spontaneous synaptic activity in the neurons from FXS patient-derived ESCs^24^. These results demonstrate the value of patient-derived ESCs and iPSCs as a model system to study molecular mechanisms of FXS.

To investigate the function of FMRP in the human nervous system, we created two *FMR1*-null human NPC lines, one from a normal human iPSC line and the other from the previously established human NPC line ReNcell CX. While these cells do not exactly represent the molecular state in patient-derived neural progenitors, they are suitable for studying the functions of FMRP, and are comparable in this regard with *Fmr1*-knockout mouse neurons. Here we report that FMRP-deficient human NPCs display altered gene expression and impaired differentiation, which is consistent with previous data from patient-derived cells. Moreover, we found that the protein kinase inhibitor LX7101 reversed the aberrant phenotype of FMRP-deficient NPCs and neurons, and discuss putative molecular targets of LX7101 and its relationship to the pathological mechanism of FXS.

## Results

### FMRP-deficient human NPCs show aberrant gene expression

**Figure 1:**
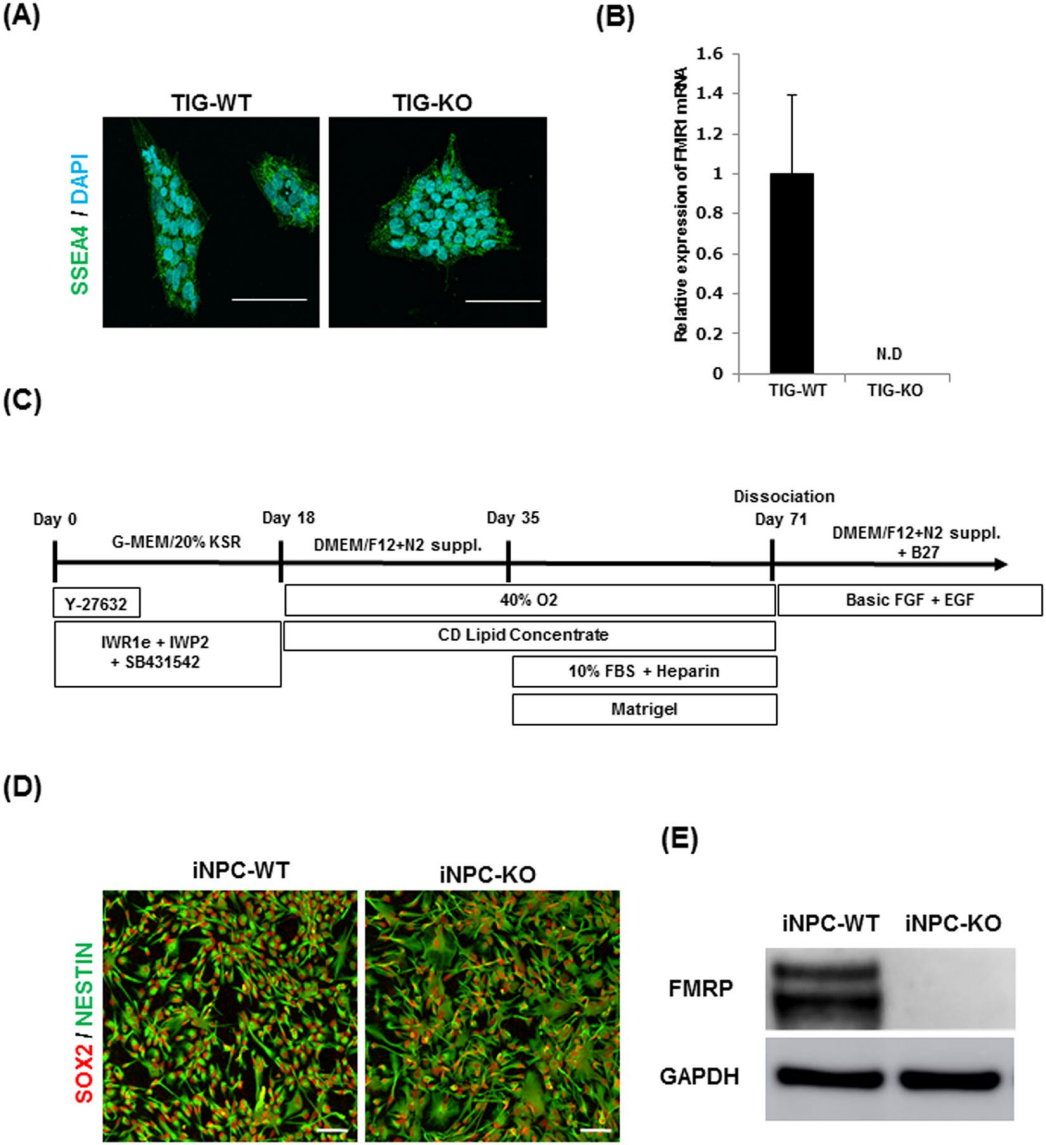
Establishment of FMRP-deficient human NPC lines. **(A)** Representative immunofluorescence staining of SSEA-4 (green) in TIG-WT and TIG-KO cells. Nuclei (blue) were stained with DAPI. Scale bars represent 100 μm. **(B)** Relative *FMR1* mRNA levels were determined using qRT-PCR analysis. The expression of *FMR1* was normalized to that of *GAPDH* in each cell clone. The *FMR1* mRNA level in TIG-WT cells was arbitrarily assigned as 1. The data are presented as mean ± S.D. of three independent experiments. N.D.: not detected. **(C)** Schematic illustration of differentiation conditions for NPC from iPSC. **(D)** Representative immunofluorescence staining of the expression of SOX2 (red) and NESTIN (green) in iNPC-WT and iNPC-KO lines. Scale bars represent 100 μm. **(E)** Western blot analysis of FMRP protein levels in iNPC-WT and iNPC-KO lines. GAPDH was used as a loading control. The representative images in replicated experiments (n = 2) were cropped and shown in this figure. Whole gel images are presented in Supplementary Fig. S1D.

To study the function of FMRP during the early stage of human neural development, we disrupted *FMR1* gene in the male iPSC line TIG114^28^. We confirmed similar expression of the stemness marker stage-specific embryonic antigene-4 (SSEA4) in both the parental (TIG-WT) and a clonal *FMR1*-KO (TIG-KO) line, and the complete absence of *FMR1* mRNA in the TIG-KO (Fig. 1A,B). There was no difference in morphology or growth between these cells. The iPSCs were then differentiated to NPCs in a serum-free floating culture of embryoid body-like aggregates with quick re-aggregation as described previously (Fig. 1C)^29^. Both the NPCs derived from TIG-WT (iNPC-WT) and TIG-KO (iNPC-KO) expressed the NPC markers SOX2 and NESTIN after day 71 (Fig. 1D), and could be stably maintained thereafter. We also tested EdU incorporation and confirmed that these cells were proliferative (Supplementary Fig. S1A–C). The absence of FMRP was also maintained (Fig. 1E).

**Figure 2:**
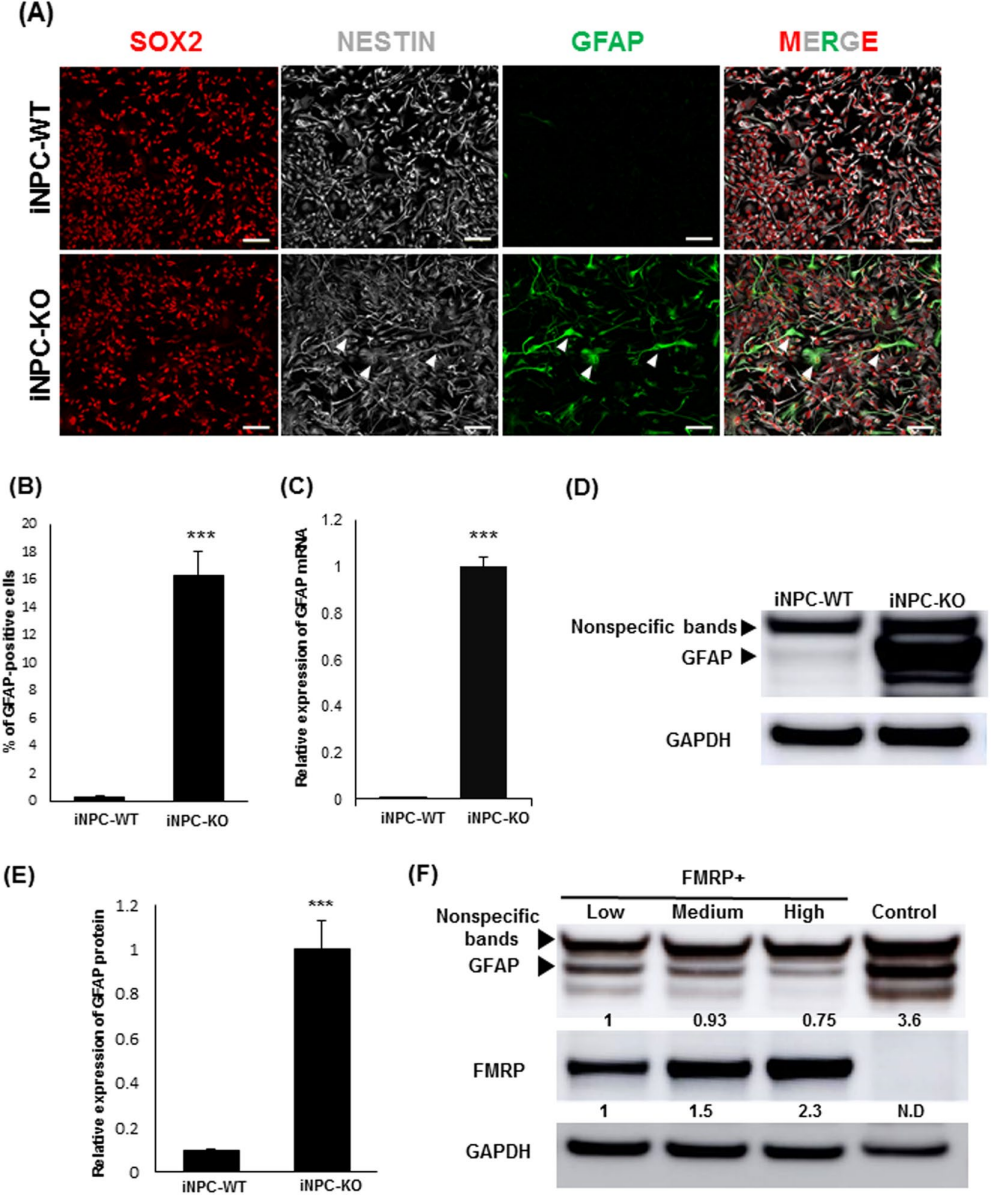
Abnormal GFAP expression in FMRP-deficient NPCs. **(A)** Representative immunofluorescence staining of the expression of SOX2 (red), NESTIN (gray) and GFAP (green) in iNPC-WT and iNPC-KO lines. Arrowheads indicate SOX2, NESTIN and GFAP-positive cells. Scale bars represent 100 μm. **(B)** Quantification of GFAP-positive cells in NPCs. The data are presented as mean ± standard deviation (S.D.) of three independent experiments (***p < 0.001). **(C)** Relative *GFAP* mRNA levels were determined using qRT-PCR analysis. The expression of *GFAP* was normalized to that of *GAPDH* in each cell line. The *GFAP* mRNA level in iNPC-KO line was arbitrarily assigned as 1. The data are presented as mean ± S.D. of three independent experiments (***p < 0.001). **(D)** Western blot analysis of GFAP protein levels in iNPC-WT and iNPC-KO lines. GAPDH was used as a loading control. The representative images in replicated experiments (n = 3) were cropped and shown in this figure. Whole gel images are presented in Supplementary Fig. S2A. **(E)** The relative quantification for GFAP protein expression normalized with GAPDH. The data are presented as mean ± S.D. of three independent experiments (***p < 0.001). **(F)** Western blot analyses of GFAP and FMRP protein levels in iNPC-KO lines infected with FMRP-expressing lentiviral (low, medium and high) and control vector (control). GAPDH was used as a loading control. The relative quantification for GFAP and FMRP expression normalized with GAPDH. The protein level in iNPC-KO line expressing Low FMRP was arbitrarily assigned as 1. N.D.: not detected. The representative images in replicated experiments (n = 2) were cropped and shown in this figure. Whole gel images are presented in Supplementary Fig. S2C.

Unexpectedly, we found that 16% of the iNPC-KO cells expressed the glial marker glial fibrillary acidic protein (GFAP) (Fig. 2A,B). The aberrant expression of GFAP was confirmed at mRNA and protein levels by quantitative PCR (qPCR) and western blotting using two different antibodies (Fig. 2C–E and Supplementary Fig. S2B). The GFAP positive cells showed glia-like elongated morphology, but co-expressed SOX2 and NESTIN (Fig. 2A, arrowheads), suggesting that they are neural progenitors rather than differentiated astrocytes. To confirm the direct link between FMRP deficiency and GFAP overexpression, we re-introduced FMRP into iNPC-KOs using a lentiviral vector. As shown in Fig. 2F, the amount of GFAP decreased proportionally to the increase in FMRP expression. These results thus indicate that FMRP deficiency caused a reversible increase in GFAP expression.

**Figure 3:**
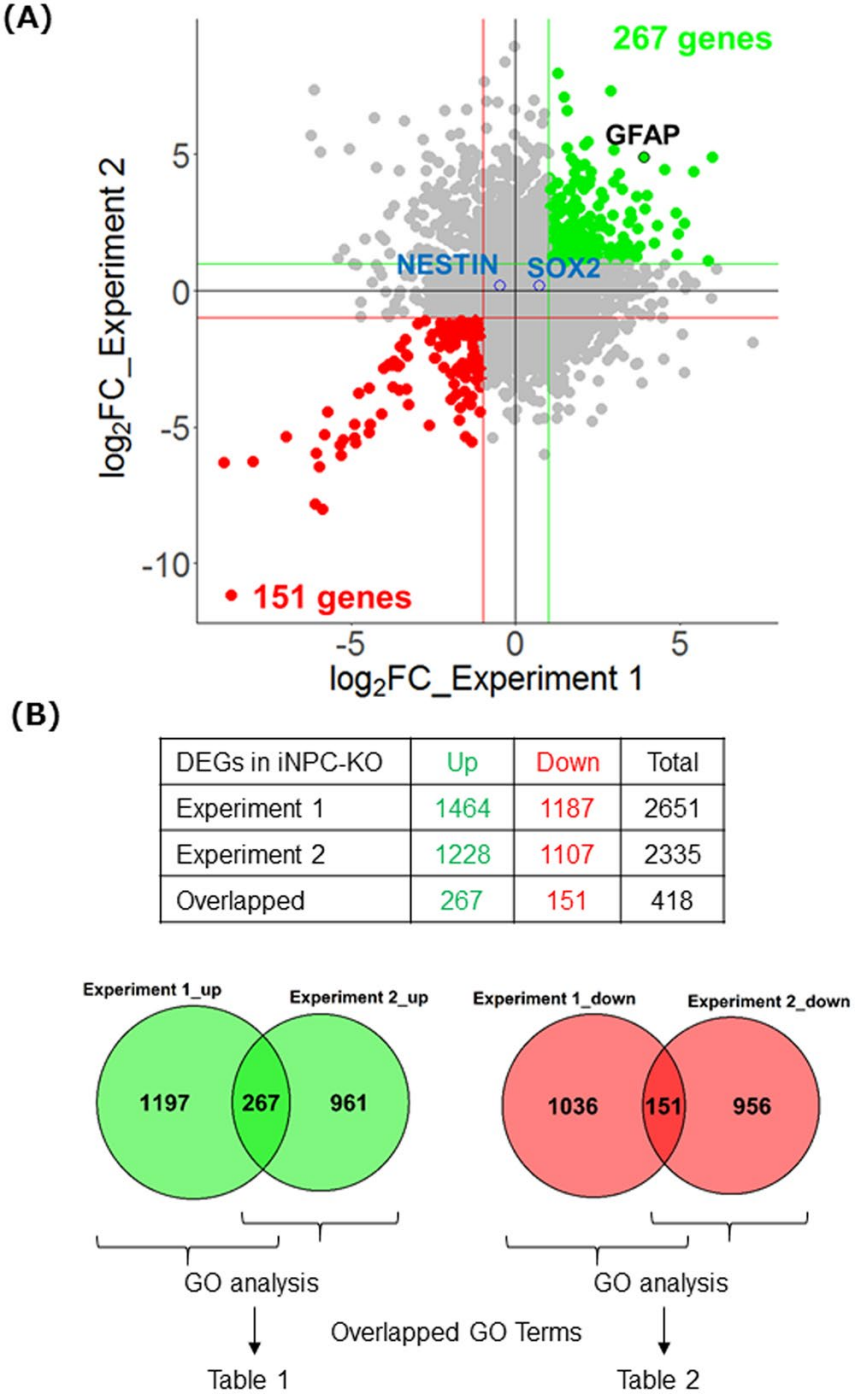
Identification and characterization of DEGs in FMRP-deficient NPCs. **(A)** Scatter plot showing the FC of each gene in two experiments. Genes with FC ≥ 2 in both experiments are colored with green (upregulated) or red (downregulated). The pluripotency markers are highlighted with blue and the glial markers with black. The horizontal and vertical lines indicate the threshold of FC = 2. (**B**) Table summarizing the number of DEGs (top) and the Venn diagrams showing the number of overlapping DEGs between two experiments (bottom left: upregulated, bottom right: downregulated). GO enrichment analyses were performed for up- or downregulated genes in each experiment, respectively. The resulting GO terms enriched in both experiments are listed in Tables 1 and 2.

We performed DNA array analysis to identify the differentially expressed genes (DEGs) in iNPC-KO. Gene expression profiling was conducted on two replicates of NPC samples that were prepared with the same protocol but in different culture lots. The expression levels of SOX2 and NESTIN in both iNPC-WT and iNPC-KO were similar, implying a similar degree of maturation to NPCs in both experiments (Fig. 3A). Although GFAP were upregulated at both experiments, neither the other glial markers (FABP7, SLC1A2, SLC1A3 and S100B, but not SPARCL1) nor neural markers (TUBB3, MAP2 and RBFOX3/NeuN) were reproducibly upregulated implying iNPC-KO was not subject to differentiate into glial or neural fate but maintained pluripotency (Fig. 3A and Supplementary Fig. S3). We defined DEGs as genes with a fold change (FC) ≥ 2 in iNPC-KO relative to iNPC-WT. The number of DEGs was similar between both experiments and several genes were reproducibly up- or downregulated (Fig. 3A,B). The number of overlapping genes, however, was relatively small (Fig. 3B). While the gene expression profiles of the two experiments were not very similar in terms of individual genes, gene ontology (GO) enrichment analysis of each experiment showed that loss of FMRP robustly affected particular biological processes. We analysed GO terms enriched in the up- or downregulated genes at each experiment and further identified the ones overlapped at the both experiments (Fig. 3B). For the upregulated genes, many GO terms associated with neural development were enriched at the both experiments (Table 1). Notably, we also observed several neural development- related GO terms in the downregulated genes that were enriched in the upregulated genes at the same time (Table 2). These result imply that iNPC neither solely obtained nor lost the tendency to differentiate into neuron but possibly show dysregulation of differentiation to neural lineages. Taken together, our results show that FMRP deficiency causes altered gene expression in NPCs, and suggest an important role for FMRP during early neural development in humans.

**Table 1:** GO terms enriched for upregulated genes in iNPC-KO.

GO Accession No.	GO Term	p-value	
		replicate1	replicate2
**GO:0007399**	**nervous system development**	**6**.**07E-12**	**7**.**14E-09**
**GO:0022008**	**neurogenesis**	**6**.**49E-09**	**4**.**66E-10**
**GO:0048699**	**generation of neurons**	**1**.**38E-07**	**1**.**50E-09**
**GO:0030182**	**neuron differentiation**	**1**.**47E-07**	**2**.**19E-08**
**GO:2000026**	**regulation of multicellular organismal development**	**2**.**58E-06**	**1**.**50E-14**
**GO:0048468**	**cell development**	**3**.**01E-05**	**5**.**14E-14**
GO:0007417	central nervous system development	6.75E-05	1.29E-04
GO:0051241	negative regulation of multicellular organismal process	9.78E-05	1.21E-09
GO:0048666	neuron development	1.01E-04	1.68E-04
GO:0051960	regulation of nervous system development	2.03E-04	3.25E-08
GO:0050767	regulation of neurogenesis	1.05E-03	1.31E-06
GO:0007166	cell surface receptor signaling pathway	1.43E-03	1.65E-13
GO:0030030	cell projection organization	1.79E-03	2.30E-06
GO:0060322	head development	2.15E-03	7.37E-03
GO:0061564	axon development	2.15E-03	1.92E-03
GO:0051240	positive regulation of multicellular organismal process	3.42E-03	2.76E-14
GO:0048812	neuron projection morphogenesis	4.76E-03	1.52E-02
GO:0031175	neuron projection development	5.31E-03	8.94E-04
GO:0048667	cell morphogenesis involved in neuron differentiation	5.69E-03	4.44E-03
GO:0007409	axonogenesis	6.72E-03	2.85E-03
GO:0060284	regulation of cell development	7.66E-03	1.31E-09
GO:0051093	negative regulation of developmental process	1.12E-02	2.29E-08
GO:0048858	cell projection morphogenesis	1.44E-02	2.68E-03
GO:0051962	positive regulation of nervous system development	1.47E-02	1.21E-06
GO:0032990	cell part morphogenesis	1.91E-02	7.04E-03
GO:0032989	cellular component morphogenesis	3.30E-02	4.14E-10
GO:0045595	regulation of cell differentiation	3.86E-02	1.22E-15
GO:0000902	cell morphogenesis	3.92E-02	9.67E-10

GO terms with statistical significance by Bonferroni correction of p value ≤ 0.05 both in experiment 1 and experiment 2 are listed. GO terms are arranged in ascending order of p-values of experiment 1. Terms appearing in both upregulated and downregulated genes are indicated in bold.

**Table 2:** GO terms enriched for downregulated genes in iNPC-KO.

GO Accession No.	GO Term	p-value	
		replicate1	replicate2
GO:0023051	regulation of signaling	1.53E-13	2.78E-02
GO:0010646	regulation of cell communication	1.62E-12	3.64E-02
**GO:2000026**	**regulation of multicellular organismal development**	**2**.**48E-09**	**1**.**53E-02**
**GO:0048468**	**cell development**	**1**.**74E-05**	**2**.**80E-05**
GO:0007267	cell-cell signaling	1.33E-04	3.30E-03
**GO:0007399**	**nervous system development**	**1**.**71E-04**	**5**.**73E-14**
**GO:0022008**	**neurogenesis**	**6**.**68E-04**	**6**.**33E-07**
**GO:0048699**	**generation of neurons**	**5**.**11E-03**	**1**.**08E-05**
GO:0045165	cell fate commitment	8.86E-03	3.55E-02
**GO:0030182**	**neuron differentiation**	**1**.**54E-02**	**3**.**44E-06**

GO terms with statistical significance by Bonferroni correction of p-value < 0.05 both in experiment 1 and experiment 2 are listed. GO terms are arranged in ascending order of p-values of experiment 1. Terms appearing in both upregulated and downregulated genes are indicated in bold.

### Protein kinase inhibitor LX7101 reverses GFAP overexpression in FMRP-deficient NPCs

**Figure 4:**
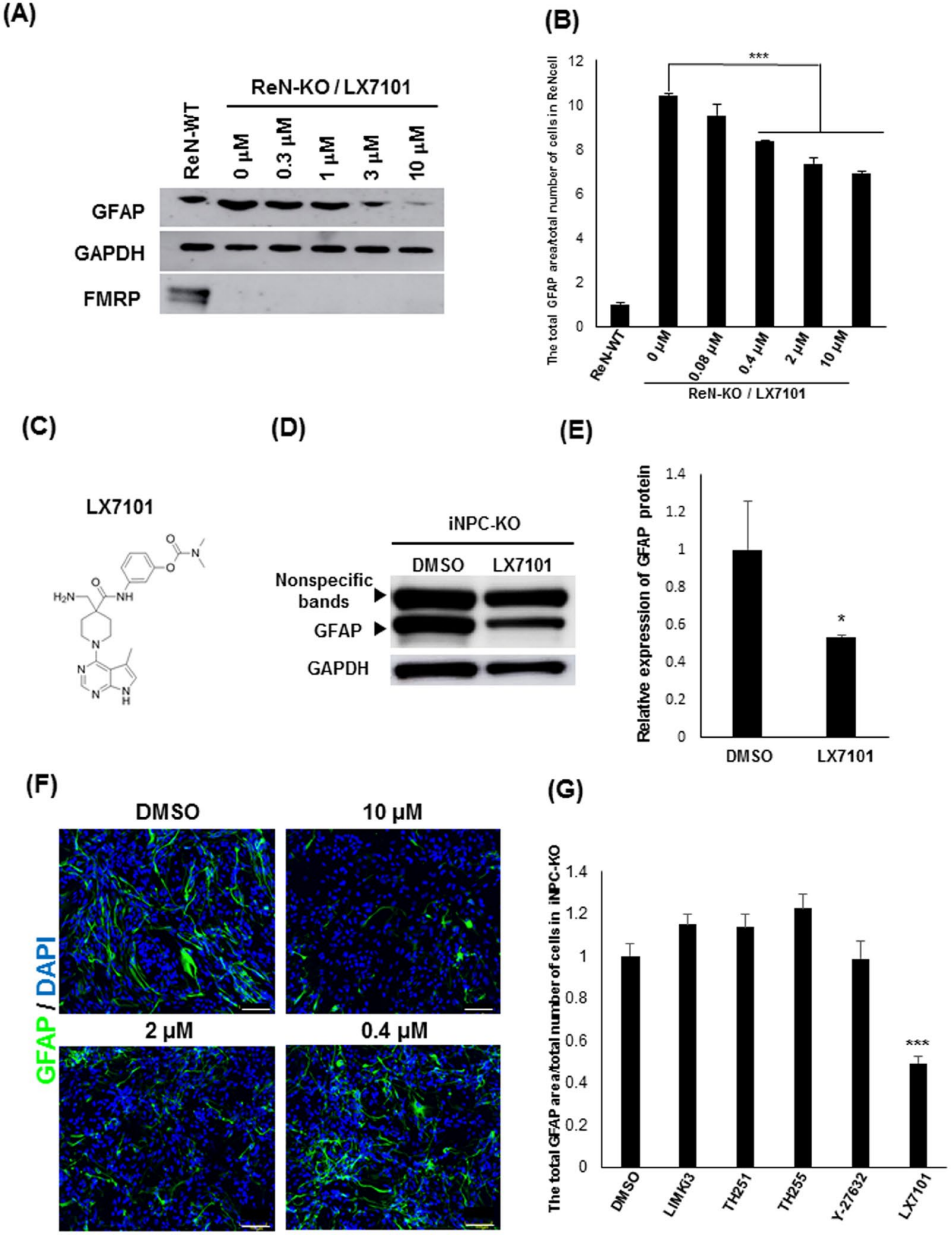
Protein kinase inhibitor LX7101 reverses GFAP overexpression in FMRP-deficient NPCs. **(A)** Representative western blot analysis of GFAP in total protein lysates of ReN-WT and ReN-KO cells 24 h after treatment with DMSO or compounds. GAPDH was used as a loading control. The representative images in replicated experiments (n = 2) were cropped and shown in this figure. Whole gel images are presented in Supplementary Fig. S4A. **(B)** Quantification of GFAP protein expression by immunofluorescence staining in ReN-WT and ReN-KO 24 h after treatment with DMSO or LX7101. The intensity of GFAP in ReN-WT was arbitrarily assigned as 1. The data are presented as mean ± S.D. of three independent experiments (***p < 0.001). **(C)** Chemical structure of LX7101. (**D**) Western blot analysis of GFAP protein levels in LX7101 and DMSO-treated iNPC-KO lines. GAPDH was used as a loading control. The representative images in replicated experiments (n = 3) were cropped and shown in this figure. Whole gel images are presented in Supplementary Fig. S4D. **(E)** The relative quantification for GFAP protein expression normalized with GAPDH. The intensity of GFAP in DMSO treated iNPC-KO was arbitrarily assigned as 1. The data are presented as mean ± S.D. of three independent experiments (*p < 0.05). (**F**) Representative immunofluorescence staining of the expression of GFAP (green) in iNPC-KO lines 72 h after treatment with LX7101 (10 μM, 2 μM, 0.4 μM) or DMSO. Nuclei (blue) were stained with DAPI. Scale bars represent 100 μm. **(G)** Quantification of GFAP area/cell in iNPCs 72 h after treatment with DMSO or compounds (LX7101: 10 μM, LIMKi3: 10 μM, TH251: 10 μM, TH255: 10 μM, Y-27612: 10 μM). The intensity of GFAP in DMSO treated iNPC-KO was arbitrarily assigned as 1. The data are presented as mean ± S.D. of three independent experiments (***p < 0.001 relative to DMSO treated iNPC).

Next, we asked if a small molecular weight compound could modify the altered phenotype of iNPC-KO. We used the immortalized human NPC line ReNcell CX^30^ to create a *FMR1*-KO cell line (ReN-KO) with the same protocol used for creating the TIG-KO line. As expected, increased expression of GFAP was also observed in ReN-KO (Fig. 4A,B). ReN-KO were suitable for screening molecular compounds because these cells have advantages of growth and easy handling compared to iPSC lines. Screening of compounds in ReN-KO identified a protein kinase inhibitor, LX7101 (Fig. 4C)^31^, that suppressed the expression of GFAP dose-dependently in concentrations between 0.3 and 10 µM (Fig. 4A,B). LX7101 was also effective in iNPC-KO in the same concentration range (Fig. 4D–F, Supplementary Fig. S2B and S4B). LX7101 at 10 µM decreased the ratio of GFAP-positive cells within 72 h of incubation (~1%), but did not affect cell growth (Supplementary Fig S4C). As LX7101 is known as an inhibitor of LIM kinase 1 (LIMK1), LIMK2, and Rho-associated protein kinase (ROCK)^31^, we tested known inhibitors for those kinases^32^ Y-27632, LIMKi3, TH251, and TH255, but found no effect (Fig. 4G). The results confirm the reversibility of GFAP overexpression in FMRP-deficient NPCs, and further suggest the possibility of correcting the aberrant phenotype by treatment with a small molecule protein kinase inhibitor.

### Spontaneous calcium bursts decrease in FMRP-deficient neurons

**Figure 5:**
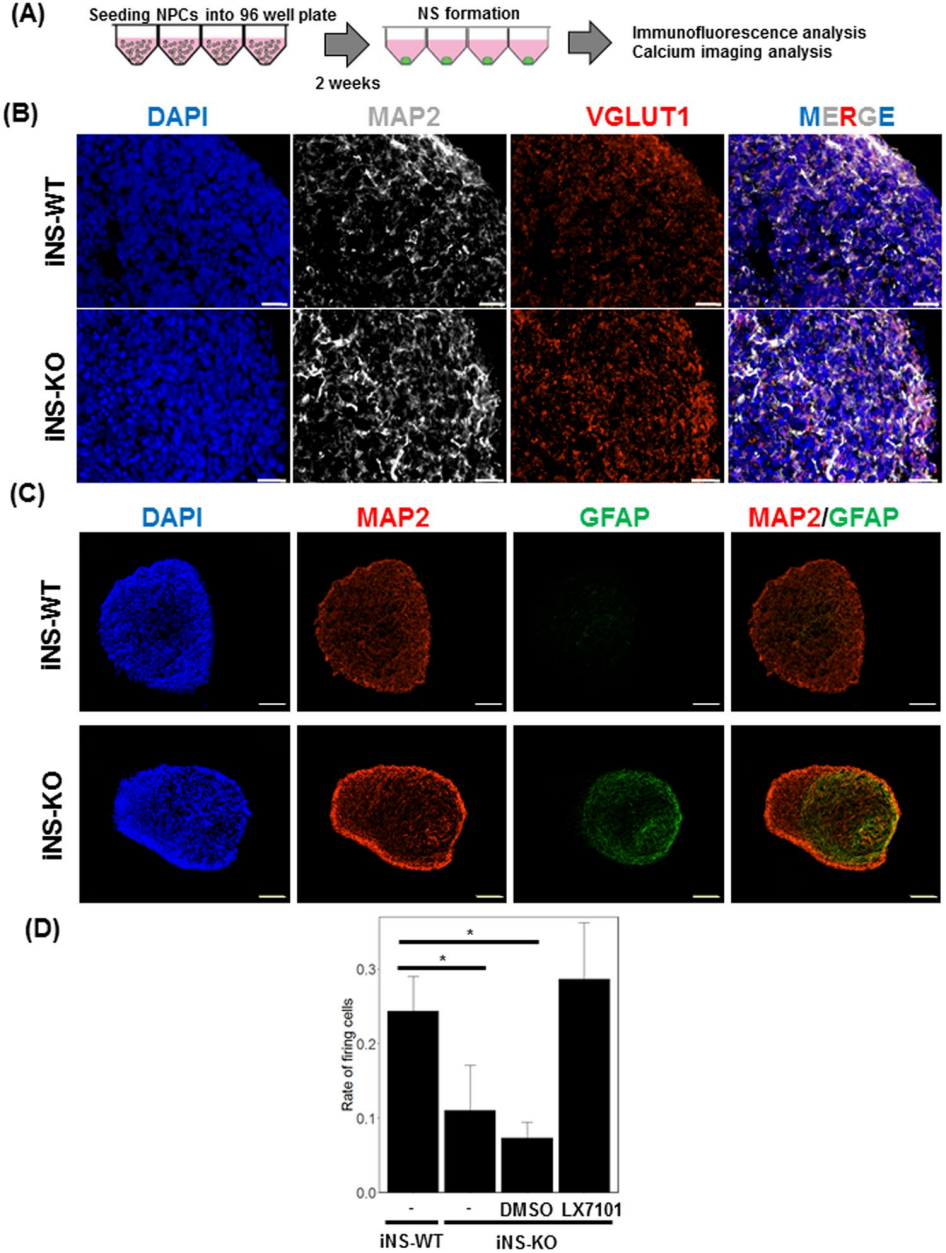
Functional characterisation of neural spheroids derived from the FMRP-deficient NPCs. **(A)** Schematic illustration of the NS formation from iNPCs. Medium with LX7101 or DMSO was changed every three days. **(B)** Representative immunofluorescence staining of MAP2 (gray) and VGLUT1 (red) in iNS-WT and iNS-KO lines. Nuclei (blue) were stained with DAPI. Scale bars represent 20 μm. **(C)** Representative immunofluorescence staining of MAP2 (red) in iNS-WT and iNS-KO lines. Nuclei (blue) were stained with DAPI. Scale bars represent 100 μm. **(D)** The rates of cells that exhibited at least one firing during the observation period. The data are presented as mean ± S.D. of three independent experiments (*p < 0.05 relative to iNS-WT).

As genes involved in neural development were widely affected in iNPC-KO, we analysed neurons differentiated from iNPC-WT and iNPC-KO. Dissociated NPCs were aliquoted in 96-well plates to allow aggregation and cultured in medium depleted of EGF and basic FGF and supplemented with the γ-secretase inhibitor DAPT (Fig. 5A). After two weeks, NPCs had differentiated into MAP2-positive neurons and formed neural spheroids (NS) (Fig. 5B). Both iNPC-WT-derived NS (iNS-WT) and iNPC-KO-derived NS (iNS-KO) expressed MAP2 and vesicular glutamate transporter 1 (VGLUT1), revealing most of the cells as glutamatergic neurons, as reported for this protocol previously (Fig. 5B)^29^. This indicates that FMRP is not essential for neuronal differentiation itself.

Abnormal GFAP expression, however, still persisted in iNS-KO (Fig. 5C). We examined the neuronal activity of NS by calcium imaging. Increase of intracellular calcium is a well-validated proxy of neuronal activation or firing, and previous reports showed that spontaneous firings of neurons developing *in vitro* can be observed as the transient bursts or oscillations of calcium^33,34^. We found spontaneous calcium transients in both iNS-KO and iNS-WT, but the firing activity was significantly lower in iNS-KO (Fig. 5D and Supplementary Movies A–D). When iNS-KO were differentiated in the presence of 10 µM of LX7101, GFAP expression was significantly reduced, and the spontaneous firing activity recovered to the level of iNS-WT. These findings suggest that FMRP is required for proper development of neuronal activity during differentiation from progenitors. In addition, protein kinase inhibition could rescue the impaired firing in FMRP-deficient NPCs.

### LX7101 affects protein phosphorylation in the AKT-mTOR pathway

**Figure 6:**
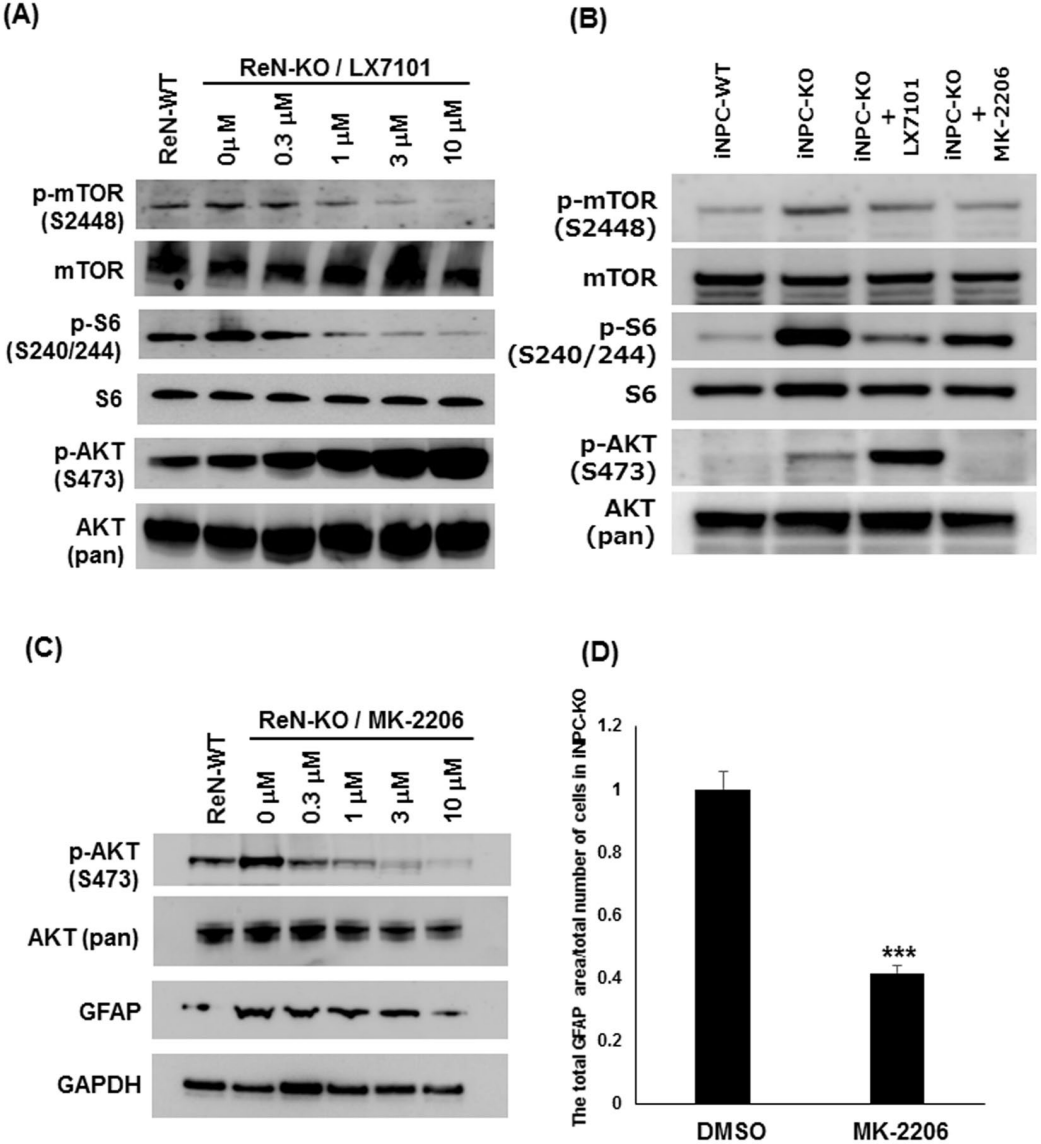
LX7101 affects protein phosphorylation in the AKT-mTOR pathway. **(A)** Western blot analysis of AKT signalling pathways in ReN-WT, DMSO-treated and LX7101-treated ReN-KO lines. The representative images in replicated experiments (n = 2) were cropped and shown in this figure. Whole gel images are presented in Supplementary Fig. S5A. (**B**) Western blot analysis of AKT signalling pathways in iNPC-WT, DMSO-treated and LX7101-treated iNPC-KO lines. The representative images in replicated experiments (n = 2) were cropped and shown in this figure. Whole gel images are presented in Supplementary Fig. S5B. (**C**) Western blot analysis of p-AKT (S473) and pan-AKT protein levels in ReN-WT, DMSO-treated and MK-2206-treated iNPC-KO lines. GAPDH was used as a loading control. The representative images in replicated experiments (n = 2) were cropped and shown in this figure. Whole gel images are presented in Supplementary Fig. S5C. (**D**) Quantification of GFAP area/cell in iNPCs 72 h after treatment with DMSO or MK-2206. The intensity of GFAP in DMSO treated iNPC-KO was arbitrarily assigned as 1. Error bars represent ± S.D. of three independent experiments (***p < 0.001).

To address the molecular mechanism of the phenotypic reversion by LX7101, we examined protein phosphorylation by western blotting after 72 h treatment with 10 µM of LX7101. LX7101 treatment reduced phosphorylation of mTOR (Ser2448) and ribosomal protein S6 (Ser240/244) in ReN-KO and iNPC-KO (Fig. 6A,B and Supplementary Fig. S5D), implying that LX7101 inhibits the AKT-mTOR pathway. Notably, phosphorylation of AKT (Ser473) was markedly increased by LX7101 in both ReN-KO and iNPC-KO (Fig. 6A,B). This paradoxical increase of AKT phosphorylation by inhibition of the AKT pathway has been reported previously^35,36^. We assumed that inhibition of AKT might suppress the overexpression of GFAP in FMRP-deficient NPCs, and indeed found that MK-2206, a highly selective allosteric inhibitor of AKT, suppressed GFAP expression (Fig. 6C,D). In addition, we found that phosphorylation of mTOR and S6 increased in ReN-KO and iNPC-KO compared with levels in the wild type NPCs (Fig. 6A,B). These findings suggest that the aberrant phenotype of FMRP-deficient NPCs might be linked to dysregulated AKT-mTOR pathway.

## Discussion

While much previous work using *Fmr1*-KO mice has focused on the FMRP-targeted protein translation at synapses, FMRP could play a role for proper neural development when it is abundantly expressed. For example, FMRP may widely affect gene expression during neurogenesis via translational control of epigenetic and transcriptional regulators^37^. We created FMRP null iPSCs to probe the physiological role of FMRP during the earliest phase of human neural development. Similar approaches using FXS patient-derived iPSCs or ESCs have been reported recently, which provided some mixed results^23–27^. Although every study reported defects in neurogenesis, there was little overlap in changes of gene expression which may be due to intrinsic differences between the respective cell lines^26,27^. All cell lines obtained from FXS patients show varying levels of *FMR1* gene expression, and control wild type cells were obtained from different individuals as well. The unique feature of our platform enables us to compare cells with and without FMRP on a more similar background.

One of the proteins affected most by the loss of FMRP in our study was GFAP. GFAP is not a binding target of FMRP, and no other direct link between GFAP and FMRP is known. We assume that the induction of GFAP expression in the FMRP null NPCs is a marker of altered differentiation representing an abnormal developmental status of the NPCs. Interestingly, Luo *et al*. reported that adult NPCs derived from *Fmr1*-KO mice or acute suppression of *Fmr1* in wildtype NPCs resulted in higher expression of GFAP upon differentiation^38^. Moreover, the group further reported that ablation of Fmrp in Nestin-positive adult NPCs impaired neurogenesis and hippocampus-dependent learning in mice accompanied by a marked increase of Gfap-positive cells in the dentate gyrus^39^. They also showed that restoration of Fmrp in the adult NPCs normalized the number of Gfap-positive cells and rescued learning deficits. Taken together, improper differentiation of FMRP null NPCs may be a common pathological mechanism underlying FXS.

We found that loss of FMRP resulted in abnormal differentiation accompanied by impaired neuronal activity, which is consistent with the results from studies using patient-derived iPSCs^24,27^. It is seemingly contradictory to the well-known concept that hyper-excitability is the primary contributor to the FXS pathology^40^. However, evidence supporting this concept has mostly come from research on *Fmr1* KO mice where nervous systems is already established. On the other hand, our study focuses on a much earlier stage of development. Indeed, it was recently reported that *Fmr*1 KO rats showed sensory hypo-excitability before eye-opening, whereas hyper-excitability emerged at later ages during the third and fourth post-natal weeks^41^. Thus, the human NPC model could be a useful tool to study previously uncharacterized functions of FMRP in the early development of nervous systems.

Another key finding of our study is that the protein kinase inhibitor LX7101 ameliorates the phenotypes of the FMRP-deficient human NPCs and neurons. The FMRP-deficient ReNcell CX cell line ReN-KO provides a robust, highly reproducible assay platform to screen compounds as well as to investigate intracellular mechanisms. To our knowledge, this is the first report of a small molecule that modulates the phenotype of FMRP-deficient human neuronal cells. LX7101 is known as a potent inhibitor for LIMK1, LIMK2, and ROCK^31^. Recently, Kashima *et al*. reported that inhibitors for LIMK1 (LIMKi3, TH251, and TH255) ameliorated synaptic and behavioural abnormalities in the mouse and *Drosophila* models of FXS^32,42^. They revealed a new mechanism that links the loss of FMRP to the impairment of synapse remodelling via an augmentation of type 2 bone morphogenic protein receptor (BMPR2) and LIMK1. They suggest that increased BMPR2 signalling activates LIMK1 and its downstream cofilin phosphorylation, which causes impairment of actin remodelling at synapses. We confirmed that all the LIMK1 inhibitors including LX7101 inhibited cofilin phosphorylation, but only LX7101 could correct the aberrant expression of GFAP. In addition, BMPR2 inhibitor LDN-193189 and ROCK inhibitor Y-27632 did not suppress the GFAP expression in our assay (Fig. 4G and Supplementary Fig. S6). It is likely that LX7101 is effective in the assays described by Kashima *et al*., because LX7101 is a highly potent inhibitor for LIMK1 and actually inhibited cofilin phosphorylation in human NPCs. Nevertheless, our data suggest that LX7101 targets a different pathway from the downstream of BMPR2 to reverse the aberrant phenotype of the FMRP-deficient human NPCs. First, LX7101 inhibited the phosphorylation of mTOR and ribosomal protein S6, both of which are downstream effectors of the AKT pathway. Second, AKT phosphorylation was increased by LX7101 treatment, which is a known consequence of AKT inhibition^35,36^. Third, another highly selective allosteric inhibitor of AKT, MK-2206, also suppressed GFAP expression.

We found that LX7101 rescue alteration of the spontaneous calcium transits in iNS-KO (Fig. 5D). In this study, the LX7101 had been present continuously during neural differentiation and maturation, and we can not deny the possibility that the LX7101 affected neuronal morphologies and functions. Whether LX7101 actives differentiation of iNS-KO into neurons or modified the functionalities of neurons (e.g.: the connectivity or length of neurites) remains to be elucidated.

Dysregulation of PI3K-AKT-mTOR signalling in FXS pathology is well-documented. For example, in the *Fmr1* KO mouse, sustained activation of PI3K was correlated with exaggerated protein synthesis and increased dendritic spine density^43^. Moreover, increased phosphorylation of mTOR and other components of the pathway was observed in the hippocampus of the *Fmr1* KO mouse, as well as in the lysate from lymphocytes or brain tissue of FXS patients^44,45^. In our study, the loss of FMRP was associated with increased phosphorylation of mTOR and S6 in both iNPC-KO and ReN-KO. On the other hand, AKT phosphorylation was markedly suppressed in iNPC-KO, but increased in ReN-KO. The reason for this difference remains to be determined. Since mTOR activation is downstream of several stimuli, feedback regulation of AKT phosphorylation may be different within those cell lines.

In summary, our findings in human NPCs are in line with the previous observations from the FXS animal models and patient-derived cells, and reveal an additional important role for FMRP during neural development. As LX7101 seems to inhibit both BMPR2-LIMK1-cofilin and AKT-mTOR pathways, it might be a new lead for novel FXS therapeutics.

## Materials and Methods

### Cell lines, media and reagents

iPSCs (TIG114-4f1) were obtained from JCRB Cell Bank. iPSC lines were maintained with StemFit medium (AK02, Ajinomoto, Tokyo, Japan).

We differentiated iPSC lines into NPCs using the SFEBq method^29^. iPSC lines were dissociated to single cells in TrypLe Express (Thermo Fisher Scientific, Waltham, MA, USA), and quickly reaggregated using Sumilon PrimeSurface plates (MB-X9901, Sumitomo Bakelite, Tokyo, Japan) in differentiation medium consisting of Glasgow-MEM (Thermo Fisher Scientific) supplemented with 20% KSR (Thermo Fisher Scientific), 0.1 mM nonessential amino acids (Thermo Fisher Scientific), 1 mM sodium pyruvate (Sigma-Aldrich, St. Louis, Missouri, USA), 0.1 mM 2-mercaptethanol and penicillin-streptomycin (Thermo Fisher Scientific). Defining the day on which the SFEBq culture was started as day 0, IWR1e (Merck, Darmstadt, Germany), IWP2 (Merck), Y-27632 (Cayman Chemical, Ann Arbor, Michigan, USA), and SB431542 (Stemgent, MA, USA) were added to culture to reach 3, 2, 10 and 5 μM, respectively, from day 0 to 5, and then reaggregates were maintained in the medium without Y-27632 from day 5 to 18. At day 18, the floating aggregates were transferred from a Sumilon PrimeSurface plate to a 6-cm Petri dish (non-cell adhesive) and further cultured in suspension with DMEM/F-12 medium supplemented with N2 (Thermo Fisher Scientific), Chemically Defined Lipid Concentrate (Thermo Fisher Scientific) and Anti-Anti (Thermo Fisher Scientific) under 40% O_2_/5% CO_2_ conditions. From day 35, 10% FBS, 5 μg/ml heparin and 1% Matrigel (Corning, NY, USA) were also added to the medium. At day 71, the aggregates were dissociated using the papain dissociation system (Worthington Biochemical Corp, Freehold, NJ) and then plated onto 6-cm cell culture dishes coated with iMatrix-511 silk (Nippi, Tokyo, Japan) in DMEM/F-12 medium supplemented with N2, B27 (Thermo Fisher Scientific), basic FGF (20 ng/ml; Pepro Tech, Rocky Hill, NJ) and EGF (20 ng/ml; Pepro Tech).

ReNcell CX human neural precursor cells were purchased from EMD Millipore (Billerica, MA, USA) and maintained according to manufacturer’s instructions.

The small-molecule inhibitors LX7101 (MedChem Express, Princeton, NJ, USA), LIMKi3 (Merck) and LDN-193189 (Sigma-Aldrich) were dissolved in dimethyl sulfoxide (Sigma-Aldrich).

### Establishment of *FMR1* KO Cells (TIG-KO/ReN-KO)

iPSCs were dissociated to single cells in TrypLe Express and suspended in Opti-MEM (Thermo Fisher Scientific) containing *FMR1* CRISPR/Cas9 plasmid (sc-401919 and sc-401919-HDR, 5 μg, respectively. Santa cruz biotechnology, Dallas, TX, USA), and then electroporated using NEPA21 (NEPAGENE, Chiba, Japan). After selection with puromycin (0.25 μg/ml; Sigma-Aldrich), cell colonies were picked up and then maintained in StemFit medium. ReNcells were dissociated to single cells in StemPro Accutase Cell Dissociation Reagent (Thermo Fisher Scientific) and electroporated using Nucleofector (Lonza, Basel, Switzerland) and Mouse Neural Stem Cell (NSC) Nucleofector™ Kit (Lonza) as described in the manual.

### qRT-PCR

Total RNA was isolated using RNeasy Mini Kit (QIAGEN, Hilden, Germany) and reverse transcription (RT)-PCR was performed using the RevaTra Ace qPCR RT master Mix with gDNA Remover (TOYOBO, Osaka, Japan) according to manufacturer’s instructions. cDNA was amplified using a CFX96 Real-Time system (Bio-rad, Hercules, USA) and a THUNDERBIRD Probe qPCR Mix (TOYOBO). PCR primers used for analysis were *FMR1* (#Hs.PT.58.24574749, IDT, San Jose, USA) and *GFAP* (#Hs.PT.58.1057167, IDT). The mRNA level was normalized to human *GAPDH* mRNA (#Hs.PT.39a.22214836, IDT).

### Immunofluorescence staining

Immunofluorescence staining for iNS was performed as described previously^29^. The cells were fixed with 4% paraformaldehyde at room temperature for 10 min. After two PBS washes, the cells were treated with blocking solution (0.2% Triton X-100; Bio-Rad /10% goat serum; Abcam, Cambridge, UK in PBS) at room temperature for 10 min. After two PBS washes, first antibody diluted in antibody solution (10% goat serum in PBS) was applied to the cells and then kept overnight at 4 °C. The cells were washed twice in PBS, followed by addition of the secondary antibody and DAPI diluted in antibody solution. After two PBS washes, the cells were observed by using the CQ1 (YOKOGAWA, Tokyo, Japan) or Leica DMi8 (Leica, Germany). Fluorescence intensity was measured using the CQ1 Measurement software (YOKOGAWA). Note that all immunofluorescence staining experiments were performed in parallel and, for detection, exposure and interval time was kept the same in several experiments. The first antibodies used were directed against SSEA-4 (1/500, #MAB4304, Merck), GFAP (1/1000, #130300, Thermo Fisher Scientific), NESTIN (1/200, #MAB5326, Merck), NESTIN (1/200, #PRB570c-100, BioLegend, San Diego, CA, USA) SOX2 (1/500, #PM059, MBL, Nagoya, Japan), VGLUT1 (1/2000, #135303, Synaptic Systems, Göttingen, Germany) and MAP2 (1/5000, #ab5392, Abcam). The secondary antibodies used were shown in Supplementary Table 1.

### Western blotting

In protein phosphorylation analysis, cells were maintained by culture media without insulin. After 12 h, compounds were added to cells. Cells were lysed in RIPA buffer and lysates were separated by SDS-PAGE using NuPAGE Bis-Tris Gel (Thermo Fisher Scientific) and then transferred to nitrocellulose membranes using iBlot (Thermo Fisher Scientific). After blocking with 10% skim milk in TBST (0.1% Tween 20/TBS), the membranes were blotted with first antibody diluted in Can Get Signal (TOYOBO) and then incubated at room temperature for 1 h. The membranes were washed twice in TBST, followed by addition of the secondary antibodies diluted in Can Get Signal and then incubated at room temperature for 1 h. After two TBST washes, antibody binding to the membranes was detected by Amersham ECL prime Western Blotting Detection Reagent (GE Healthcare, Piscataway, NJ, USA) and Amersham imager 600 (GE Healthcare). The first antibodies used were directed against GFAP (1/2500, #130300, Thermo Fisher Scientific, 1/1000, #53554, abcam), FMRP (1/1000, #7104, Cell Signaling Technology, Beverly, MA, USA), GAPDH (1/1000, #M171-7, MBL), mTOR (1/1000, #2983 P, Cell Signaling Technology), p-mTOR (1/1000, #5536 S, Cell Signaling Technology), AKT (1/1000, #4691 P, Cell Signaling Technology), p-AKT-S473 (1/2000, #4060 P, Cell Signaling Technology), S6 (1/1000, #2217 S, Cell Signaling Technology) and p-S6 (1/1000, #2215 S, Cell Signaling Technology). The quantification for western blot analysis were normalized to the loading control (GAPDH, panAKT, S6 and mTOR).

### DNA microarray analysis

Total RNA was extracted from iNPCs prepared as described above using the RNeasy Mini kit (QIAGEN). Purified RNA was then amplified and labelled with cyanine 3 using the one-color Low Input Quick Amp Labeling Kit (Agilent Technologies, CA, USA) according to the manufacturer’s instructions. The labelled cRNA was fragmented and hybridized to the Agilent SurePrint G3 Human GE 8 × 60 K Ver. 3.0 Microarray. After the microarrays were washed, they were scanned with an Agilent DNA Microarray Scanner. The intensity value for each scanned feature was quantified with the Agilent Feature Extraction software, which subtracted the background. Agilent GeneSpring GX version13.1 was used for normalization as follows. First, signal intensities <1.0 were set to 1.0. Each chip was then normalized to the 75th percentile of the measurements taken from that chip. Finally the normalized intensities were log2-transformed. The microarray data have been submitted to the National Center for Biotechnology Information Gene Expression Omnibus (GEO) and are available under the accession number GSE108560.

### Functional enrichment analysis

Functional annotations (GO) were performed by subjecting DEGs to the DAVID v6.7 (http://david-d.ncifcrf.gov/). GO terms with Bonferroni corrected p value ≤ 0.05 were identified as enriched functions.

### Statistical analysis

For the statistical comparisons, two-sided Student’s t test was used for comparisons between two sets of data in Figs 2B,C,E, 4E and 6D and Supplementary Figs S4B,C and S6, and Dunnett’s test was used for multiple comparisons in Figs 4B,G and 5D. Significance was established at *P*-values less than 0.05.

### Data availability

Microarray data are available under GSE108560. All other data generated or analysed during this study are included in this article and the supplementary information.

## Electronic supplementary material

Supplementary Movie A

Supplementary Movie B

Supplementary Movie C

Supplementary Movie D

Supplementary information
