# Supplementary material for: Loss of the fragile X mental retardation protein causes aberrant differentiation in human neural progenitor cells
Source: Sci Rep. 2018 Aug 2;8:11585. doi: 10.1038/s41598-018-30025-4 (PMC6072755; doi:10.1038/s41598-018-30025-4)
Supplement: Supplementary file 5 — Supplementary information [file 41598_2018_30025_MOESM5_ESM.pdf]

Loss of the fragile X mental retardation protein causes aberrant differentiation in human neural progenitor cells

Naohiro Sunamura, Shinzo Iwashita, Kei Enomoto, Taisuke Kadoshima* & Fujio Isono *

Asubio Pharma Co., Ltd., 6-4-3 Minatojima-minamimachi, Chuo-ku, Kobe 650-0047, Japan.

*Correspondence and requests for materials should be addressed to T.K. and F.I

(email: kadoshima.taisuke.tw@daiichisankyo.co.jp and isono.fujio.vd@daiichisankyo.co.jp, respectively)

Supplementary Figure S1

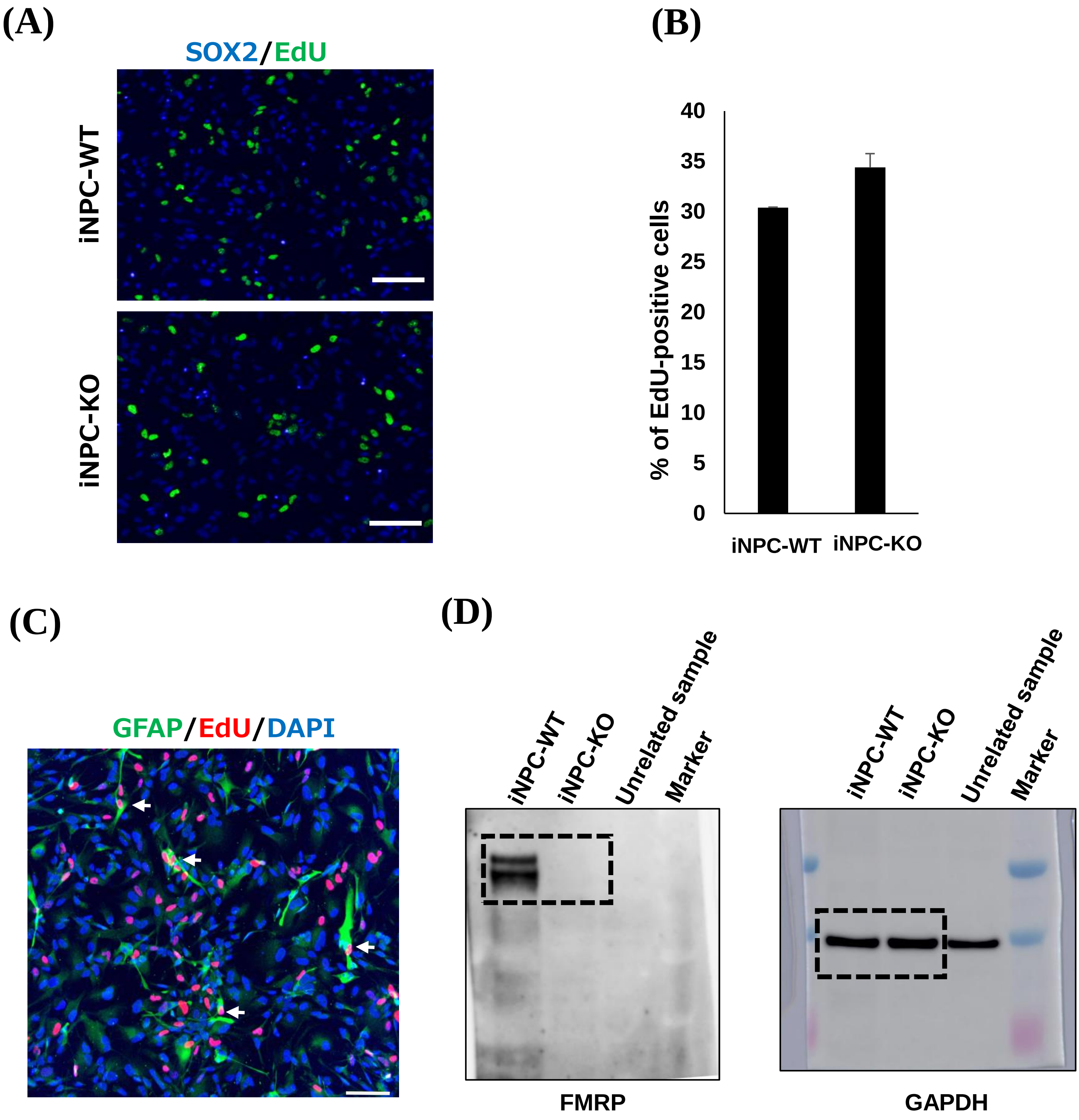

Supplementary Figure S1.

(A) Representative images of EdU-positive cells in iNPCs after EdU labelling for 3 h. Nuclei (blue) were stained with DAPI. Scale bars represent 100 µm. (B) Quantification of EdU incorporation in iNPCs. (C) Representative image of GFAP (green) and EdU (red) in iNPC-KO. Nuclei (blue) were stained with DAPI. Arrows indicate GFAP and EdU-positive cells. Scale bar represents 100 µm. (D) Whole western blots images in Figure 1E. Dots frames indicate fragments presented in the Figure 1E in the main text.

Supplementary Figure S2

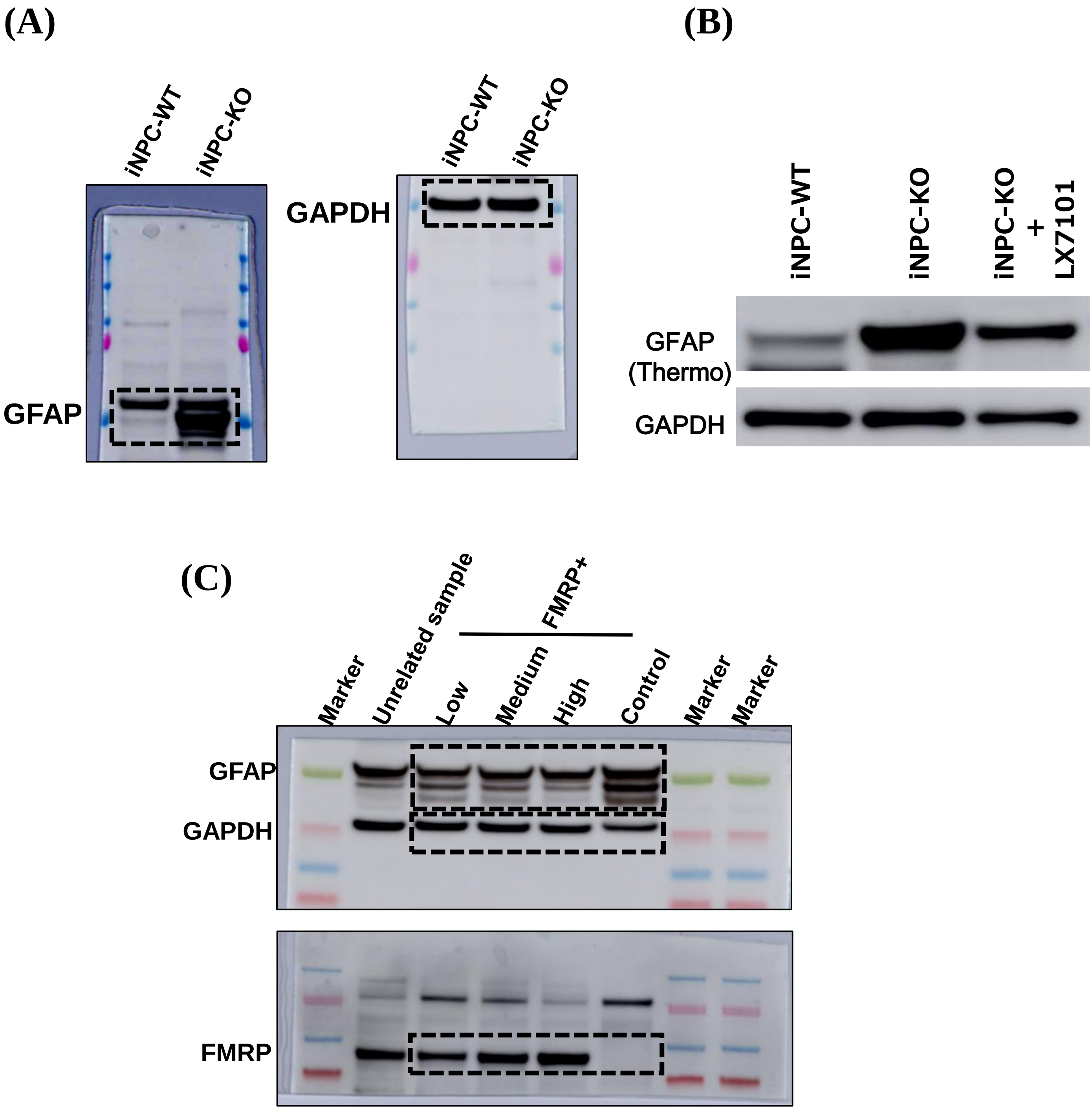

Supplementary Figure S2.
(A) Dots frames indicate fragments presented in the Figure 2C in the main text. (B) Western blot analysis of GFAP protein levels in iNPC-WT, iNPC-KO lines treated with DMSO and LX7101 using another GFAP antibody (Thermo Fisher). GAPDH was used as a loading control. Cropped blot images were used in this figure. (C) Dots frames indicate fragments presented in the Figure 2E in the main text.

Supplementary Figure S3

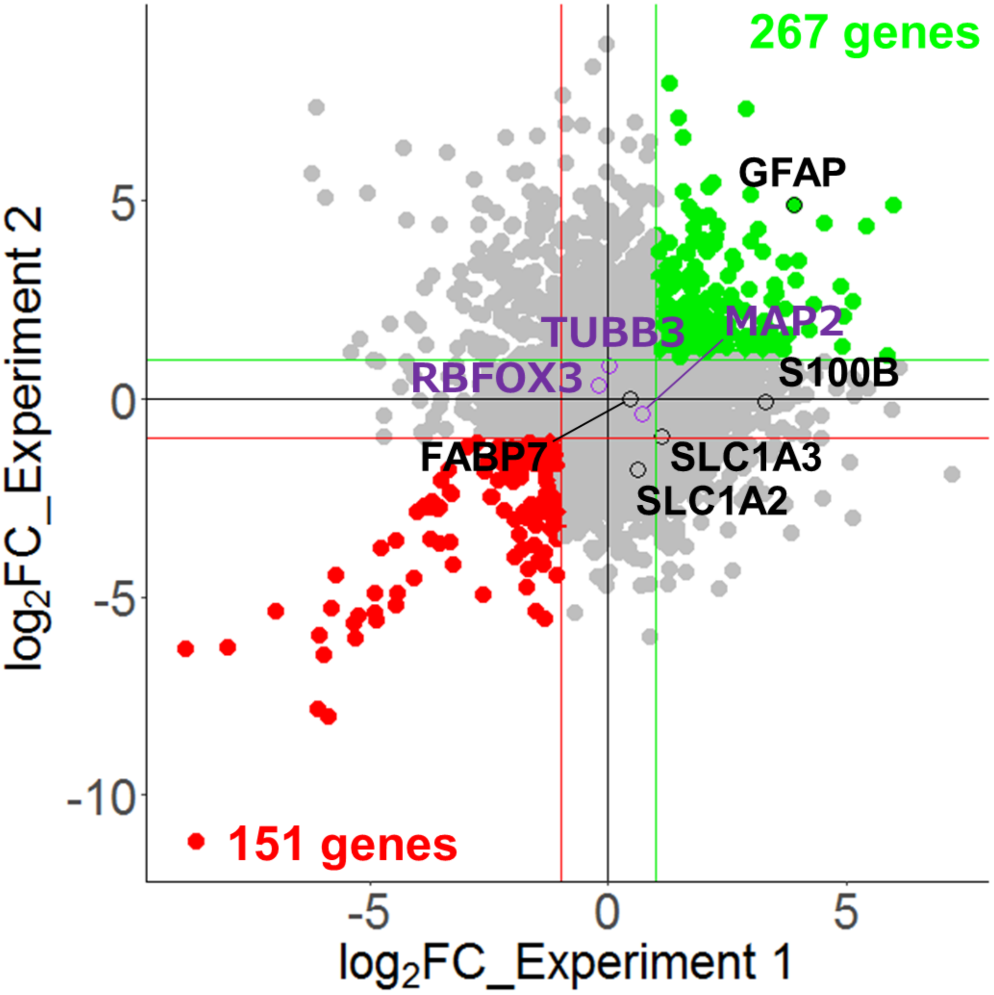

Supplementary Figure S3
Scatter plot showing the FC of each gene in two experiments. Genes with FC ≥ 2 in both experiments are colored with green (upregulated) or red (downregulated). The glial markers are highlighted with black and the neural markers with purple.

Supplementary Figure S4

(A)

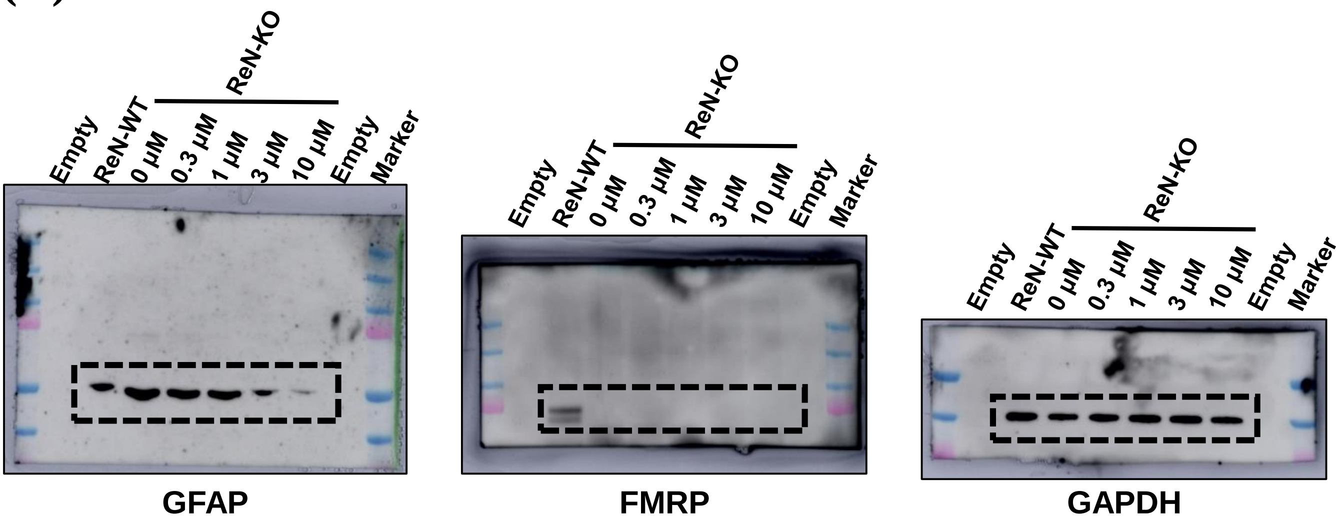

(B)

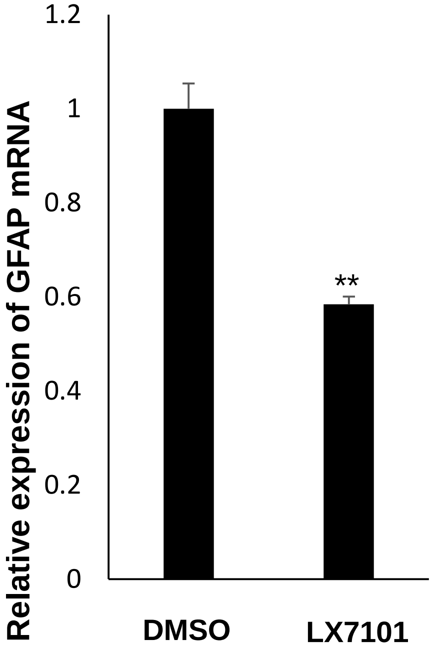

(C)

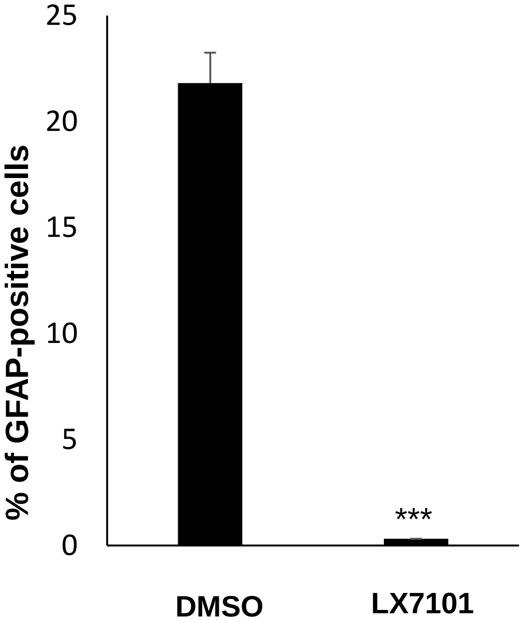

Supplementary Figure S4

(D)

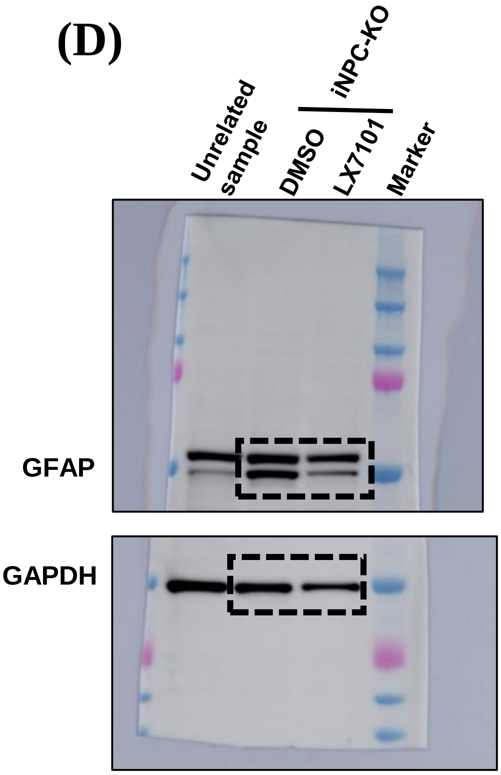

Supplementary Figure S4.

(A) Whole western blots images in Figure 4A. Dots frames indicate fragments presented in the Figure 4A. (B) Relative *GFAP* mRNA levels were determined using qRT-PCR analysis. The expression of *GFAP* was normalized to that of *GAPDH* in each cell line. The *GFAP* mRNA level in iNPC-KO line treated with DMSO was arbitrarily assigned as 1. The data are presented as mean ± S.D. of three independent experiments (**p < 0.01). (C) Quantification of GFAP-positive cells in iNPC treated with DMSO or LX7101. The data are presented as mean ± S.D. of three independent experiments (***p < 0.001). (D) Whole western blots images in Figure 4D. Dots frames indicate fragments presented in the Figure 4D in the main text.

Supplementary Figure S5

(A)

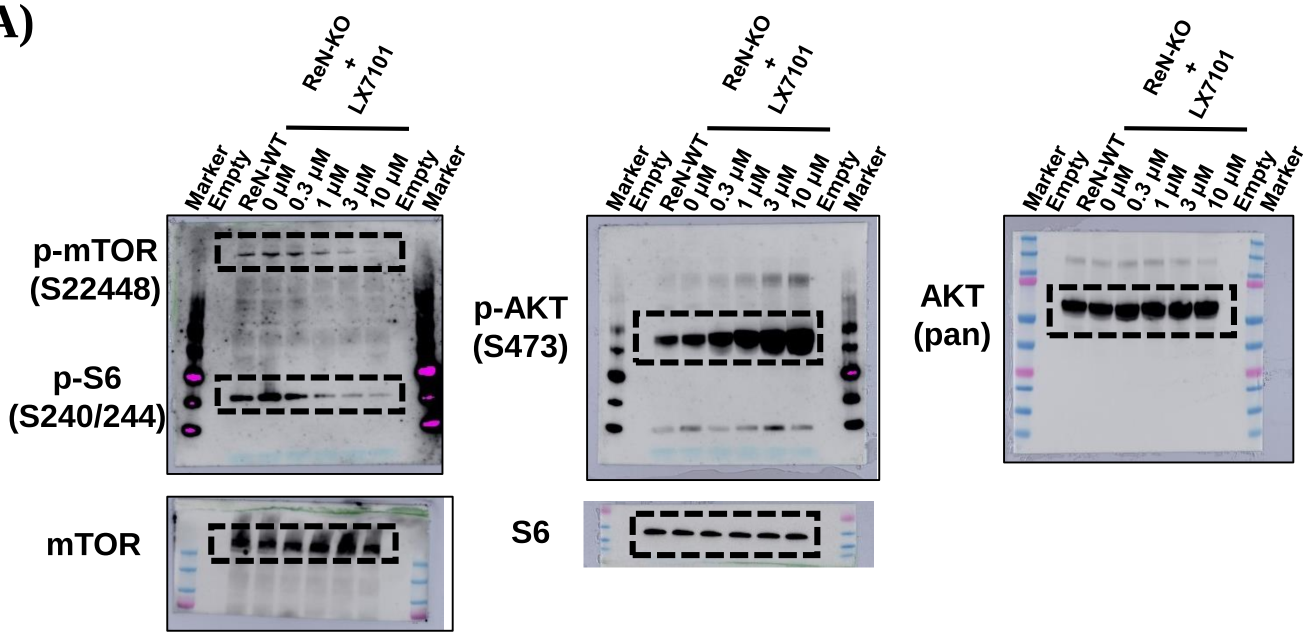

(B)

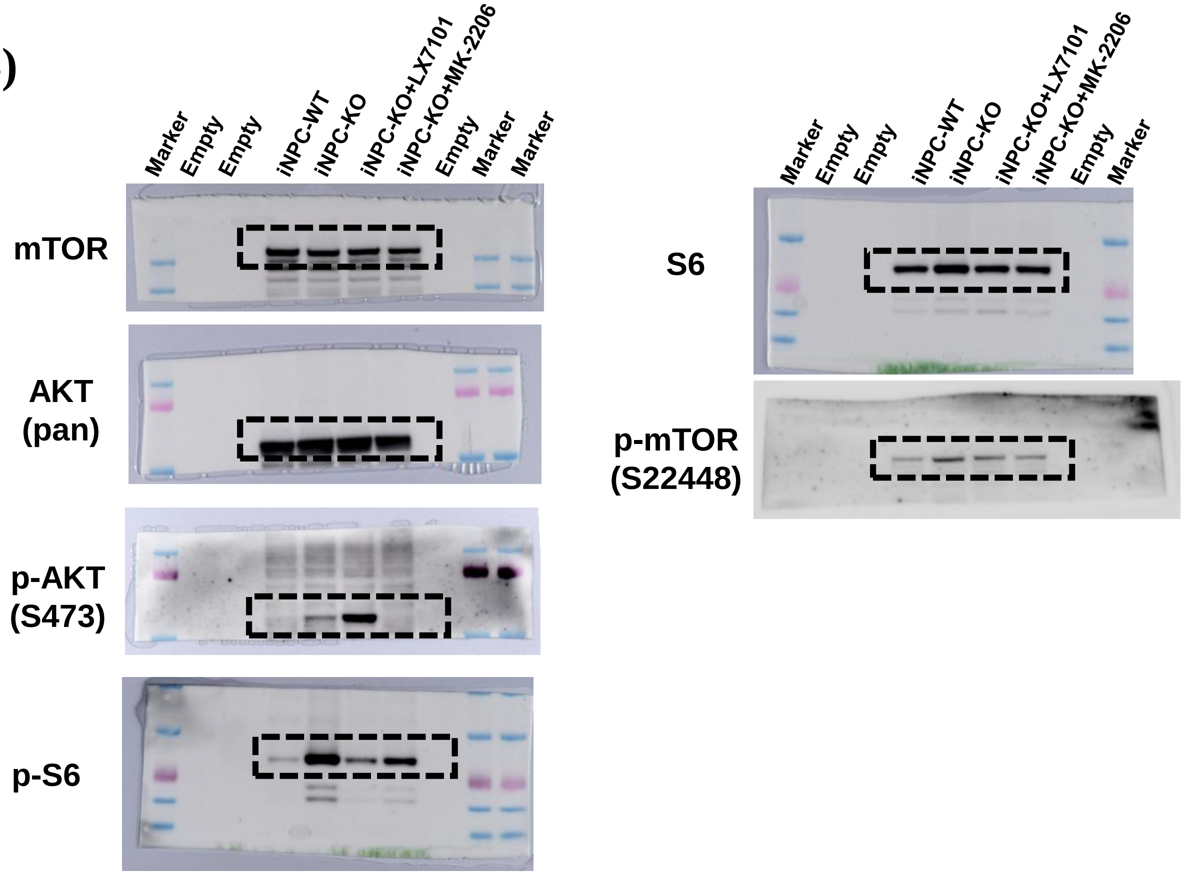

Supplementary Figure S5

(C)

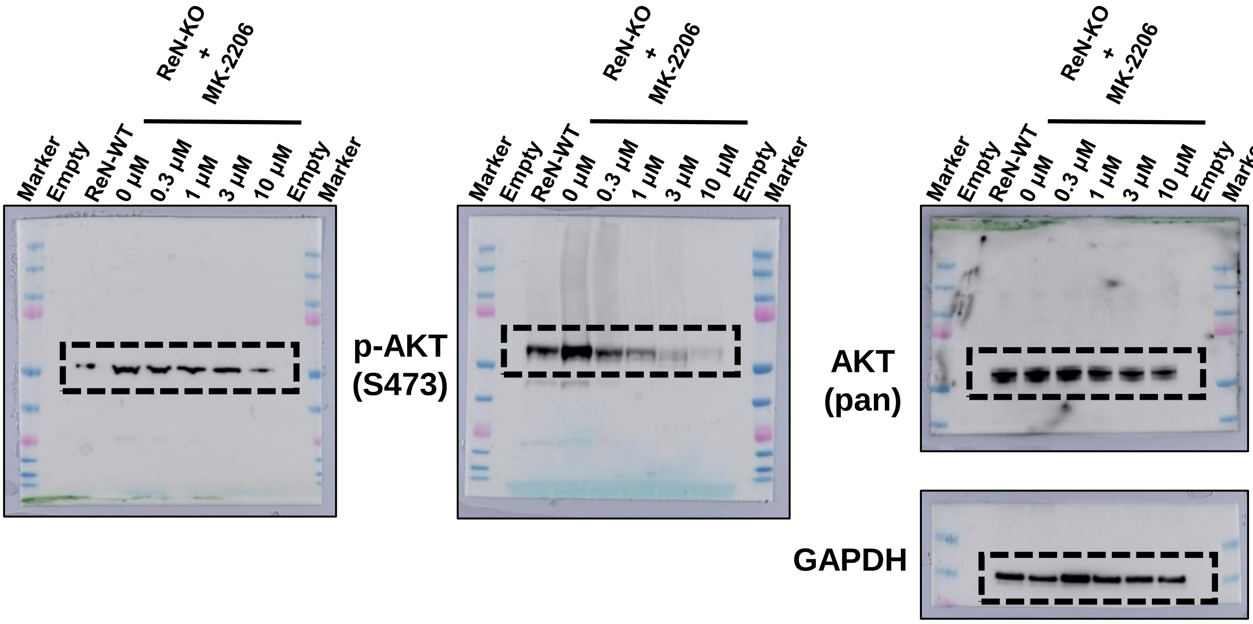

(D)

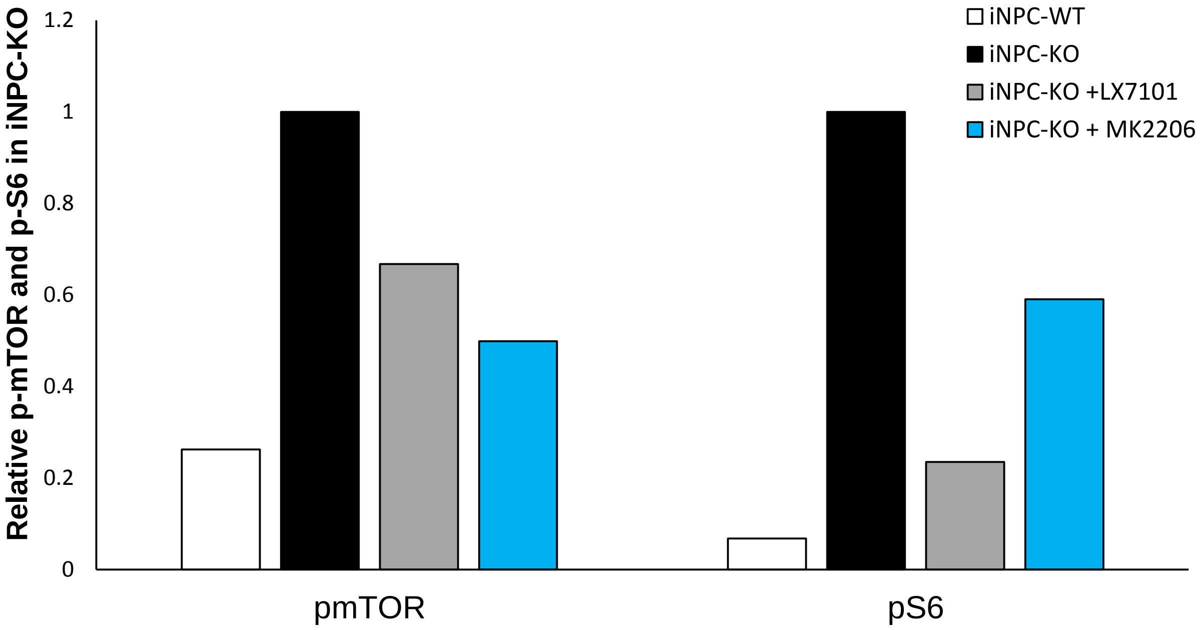

Supplementary Figure S5.
(A)-(C) Whole western blots images in Figure 6A-C. Dots frames indicate fragments presented in the Figure 6A-C in the main text. (D) Quantification of p-mTOR and p-S6 in iNPC-WT and iNPC-KO treated with LX7101 or MK-2206. iNPC-KO was arbitrarily assigned as 1.

Supplementary Figure S6

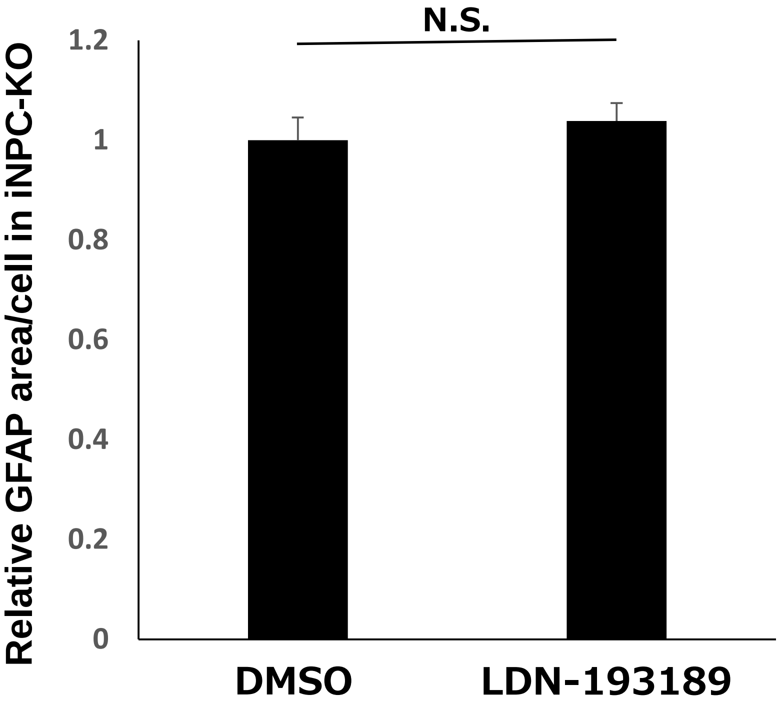

Supplementary Figure S5.

Quantification of GFAP area/cell in iNPCs 72 h after treatment with DMSO or LDN-193189. The intensity of GFAP in DMSO treated iNPC-KO was arbitrarily assigned as 1. Error bars represent ± S.D. of three independent experiments.

Materials and Methods

EdU incorporation assay

EdU incorporation assay was performed using Clik-iT EdU Alexa Fluor 488 Imaging kit (Thermo Fisher Scientific) according to the manufacturer' instructions.

Plasmid constructs and lentiviral-mediated transduction

To generate FMRP-expressing lentivirus vector, human *FMR1* (NM_001185076.1) was PCR-amplified from a synthetic DNA sequence (Eurofins Genomics, Ebersberg, Germany) and the PCR fragments were then introduced into a BamHI-digested pLVSIN-EF1α Neo vector (Clontech, Palo Alto, CA) using In Fusion Cloning kit (Clontech) according to the manufacturer's instructions. Empty vector was used as control. The PCR primer sets used were as follows: forward primer 5'- GAGCGGCCGCGGATCATGGAGGAGCTGGTGGTG, and reverse primer 5'-CGGTAGAATTGGATCTTAGGGTACTCCATTCACG. Lentivirus containing FMRP-expressing lentivirus vector or empty vector was produced in Lenti-X 293T cells (Clontech) by Lentiviral High Titer Packaging Mix (Clontech) transfected via Lipofectamine 2000 (Thermo Fisher Scientific), and supernatants containing lentivirus were concentrated with Lent-X concentrator (Clontech). Transduction of the lentivirus into iNPC-KO was performed with polybrene (Sigma-Aldrich) according to the manufacturer's instructions.

Screening and compounds treatment

The ReN-WT and KO cells (2 x 10^4 cells/well) were seeded into 96 well plates (#6005558, Perkin Elmer) and kept overnignht in CO_2 incubator. The randomly selected 20,000 compounds (10 μM) were added to the cells, and after 24 h, the cells were fixed with 4% paraformaldehyde at room temperature for 10 min. After PBS washes, these cells were used in immunofluorescence staining analysis.

Optimization of compound concentration

We pre-tested the hit-compounds at various concentrations, and optimized concentration that did not induce cell death in iNPC-KO and ReN-KO cells, followed by performing experiment. In brief, the cells (2 x 10^4 cells/well) were seeded into 96 well plates and kept overnignht in CO_2 incubator. The hit-compounds at various concentrations (20 μM-0.02 μM) were added to the cells, and after 24 h, the cells were fixed with 4% paraformaldehyde at room temperature for 10 min. We performed DAPI staining and detected the number of living cells using CQ1.

Calcium imaging and quantification of the spontaneous firing of cells in iNSs

iNPC lines were dissociated to single cells in StemPro Acctase cell Dissociation Reagent (Thermo Fisher Scientific), and reaggregated using Sumilon PrimeSurface plates (40,000 cells/well) in BrainPhys Neuronal Medium (STEMCELL technologies, Vancouver, British Columbia, Canada) supplemented with N2, B27 and 10 μM DAPT (Sigma-Aldrich). LX7101 or DMSO were present continuously by analysis (day 1-14). After two weeks, Cal-520 (AAT Bioquest, Sunnyvale, CA, USA) was added into medium and then incubated for 2 h at 37 ℃. iNS were washed twice in HHBS, and analysed using CQ1.The activities of neurons in iNS were monitored by calcium imaging for 120 sec. The signal intensities were obtained from the time-lapse calcium imaging data of arbitrary cross-sectional surface of iNS (frame rate: 1 fps). To identify the individual cells in each record, the data were analysed using image software (CQ1 software, Yokogawa). The candidate regions of interest (ROIs) were created by segmentation of pixels based on thresholding the accumulated signal intensities over observation period, which were reviewed manually to ensure correct mapping of the location of cells. Then the activities of individual ROIs were calculated. The baseline intensity (F) was estimated by the intensity of each ROI based on the exponential decay model and ⊿F/F was calculated[1]. The firing is defined as the spike with ⊿F/F > 0.2. The ROIs exhibited at least one firing during the observation period were counted as active cells and the rate of active cells were calculated as indicator of the firing activity.

Reference

1. Jia, H., Rochefort, N. L., Chen, X. & Konnerth, A. In vivo two-photon imaging of sensory-evoked dendritic calcium signals in cortical neurons. *Nature protocols* **6**, 28-35, doi:10.1038/nprot.2010.169 (2011).

Supplementary Table 1

Supplementary Table 1. List of secondary antibodies used in this study

Antibody	Catalogue No.	Dilution	Source
AlexaFluor488 goat anti-mouse antibody	#A11029	1/500	Thermo Fisher Scientific
AlexaFluor488 goat anti-rat antibody	#A11006	1/500	Thermo Fisher Scientific
AlexaFluor546 goat anti-chicken antibody	#A11040	1/500	Thermo Fisher Scientific
AlexaFluor647 goat anti-chicken antibody	#A21449	1/500	Thermo Fisher Scientific
AlexaFluor647 goat anti-mouse antibody	#A21235	1/500	Thermo Fisher Scientific
AlexaFluor546 goat anti-rabbit antibody	#A11010	1/500	Thermo Fisher Scientific
AlexaFluor488 goat anti-rabbit antibody	#A11008	1/500	Thermo Fisher Scientific
